# 3-[(*E*)-2-(4-Chloro­phen­yl)ethen­yl]-5,5-di­methyl­cyclo­hex-2-en-1-one

**DOI:** 10.1107/S1600536813016255

**Published:** 2013-06-19

**Authors:** Zeenat Fatima, Govindaraj Senthilkumar, A. Vadivel, Haridoss Manikandan, Devadasan Velmurugan

**Affiliations:** a Centre of Advanced Study in Crystallography and Biophysics, University of Madras, Guindy Campus, Chennai 600 025, India; b Department of Chemistry, Annamalai University, Annamalainagar 608 002, Tamilnadu, India

## Abstract

In the title compound, C_16_H_17_ClO, the cyclo­hexene ring adopts a half-chair conformation and the best plane through the six ring atoms makes a dihedral angle of 6.69 (7)° with the chlorophenyl ring. In the crystal, pairs of C—H⋯O hydrogen bonds link the mol­ecules into centrosymmetric *R*_2_^2^(20) dimers. The dimers are linked into an infinite chains along the *b*-axis direction by further C—H⋯O hydrogen bonds.

## Related literature

For the pharmacological activity of cyclo­hexa­none derivatives, see: Puetz *et al.*(2003 ▶); Rajveer *et al.* (2010 ▶). For a related structure, see: Hema *et al.* (2006 ▶). For conformational analysis, see: Allinger (1977 ▶); Cremer & Pople (1975 ▶).
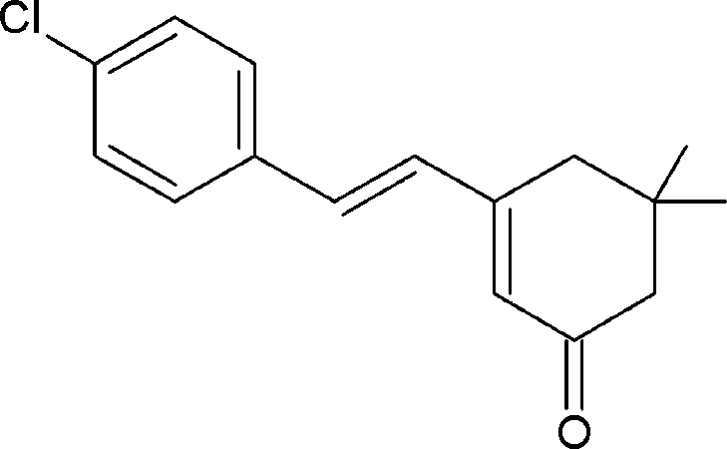


## Experimental

### 

#### Crystal data

C_16_H_17_ClO
*M* *_r_* = 260.75
Monoclinic, 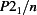

*a* = 13.7630 (4) Å
*b* = 6.0841 (2) Å
*c* = 17.5003 (6) Å
β = 105.726 (2)°
*V* = 1410.54 (8) Å^3^
*Z* = 4
Mo *K*α radiation
μ = 0.26 mm^−1^
*T* = 293 K
0.30 × 0.25 × 0.20 mm

#### Data collection

Bruker SMART APEXII area-detector diffractometer
Absorption correction: multi-scan (*SADABS*; Bruker, 2008 ▶) *T*_min_ = 0.927, *T*_max_ = 0.951
13158 measured reflections
3552 independent reflections
2611 reflections with *I* > 2σ(*I*)
*R*_int_ = 0.022

#### Refinement

*R*[*F* ^2^ > 2σ(*F* ^2^)] = 0.044
*wR*(*F* ^2^) = 0.130
*S* = 1.03
3552 reflections
165 parameters
H-atom parameters constrained
Δρ_max_ = 0.21 e Å^−3^
Δρ_min_ = −0.33 e Å^−3^

### 

Data collection: *APEX2* (Bruker, 2008 ▶); cell refinement: *SAINT* (Bruker, 2008 ▶); data reduction: *SAINT*; program(s) used to solve structure: *SHELXS97* (Sheldrick, 2008 ▶); program(s) used to refine structure: *SHELXL97* (Sheldrick, 2008 ▶); molecular graphics: *ORTEP-3 for Windows* (Farrugia, 2012 ▶); software used to prepare material for publication: *SHELXL97* and *PLATON* (Spek, 2009 ▶).

## Supplementary Material

Crystal structure: contains datablock(s) global, I. DOI: 10.1107/S1600536813016255/bt6912sup1.cif

Structure factors: contains datablock(s) I. DOI: 10.1107/S1600536813016255/bt6912Isup2.hkl

Supplementary material file. DOI: 10.1107/S1600536813016255/bt6912Isup3.cml

Additional supplementary materials:  crystallographic information; 3D view; checkCIF report

## Figures and Tables

**Table 1:** Hydrogen-bond geometry (Å, °)

*D*—H⋯*A*	*D*—H	H⋯*A*	*D*⋯*A*	*D*—H⋯*A*
C2—H2⋯O1^i^	0.93	2.41	3.301 (2)	159
C14—H14*B*⋯O1^ii^	0.97	2.57	3.4299 (18)	148

Symmetry codes: (i) 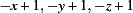; (ii) 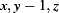.

**Fig. 1.**
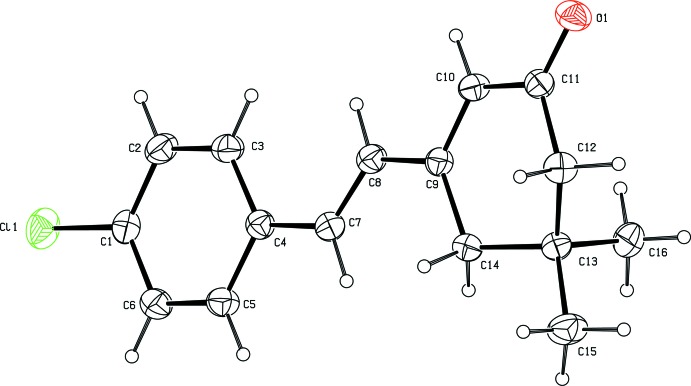
The molecular structure of the title compound, showing displacement ellipsoids drawn at the 30% probability level. H atoms are presented as small spheres of arbitrary radius.

**Fig. 2.**
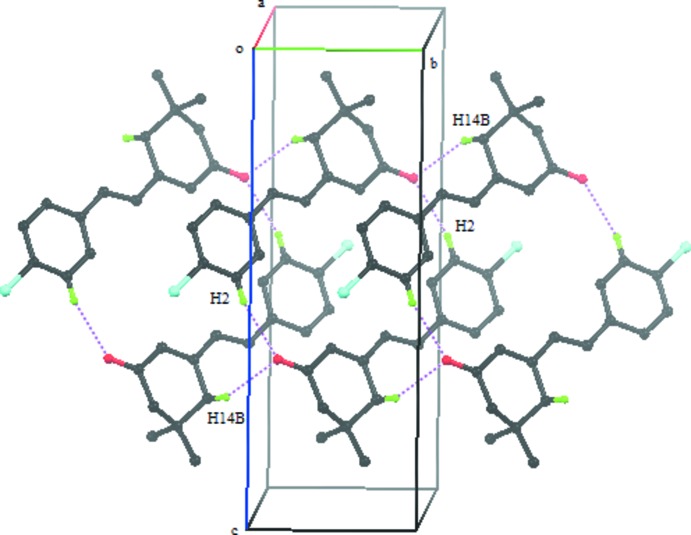
The crystal packing of the title compound viewed down a axis. H-atoms not involved in H-bonds have been excluded for clarity.

## supplementary crystallographic information

### Comment

Cyclohexanone derivatives have potent pharmacological activity in the treatment of a broad spectrum of medical conditions (Puetz *et al.*, 2003). The cyclohexanone moiety constitutes an important structural feature in several antiinflammatory, analgesic, local anesthetic and antihistaminic drugs (Rajveer *et al.*, 2010). In view of different applications of this class of compounds, we have undertaken a single-crystal structure determination of the title compound.

Molecules of the title compound, C_16_H_17_ClO, (Fig.1) are linked by pairs of intermolecular (C2—H2···O1) hydrogen bonds (Fig. 2) into centrosymmetric *R*_2_^2^(20) dimers and these dimers are linked into an infinite chains by pairs of (C14—H14B···O1) hydrogen bonds, which propagate in the *b* axis direction. The cyclohexene ring (C9—C14) adopts a *half-chair* conformation and makes a dihedral angle of 6.69 (7)° with the phenyl ring (C1—C6).

### Experimental

A mixture of isophorone (0.01 mol), 4-chlorobezaldehyde (0.01 mol) and sodium hydroxide solution (10 mL, 10%) in ethanol (25 mL) was stirred at room temperature until the starting material disappeared. The resulting mixture was poured into crushed ice and the precipitate was filtered off, dried and recrystallized from ethanol. Yield=90%, Melting point=84–86°C.

### Refinement

The hydrogen atoms were placed in calculated positions with C—H = 0.92 Å to 0.97 Å refined in the riding model with fixed isotropic displacement parameters:*U*_iso_(H) = 1.5*U*_eq_(C) for methyl group and *U*_iso_(H) = 1.2*U*_eq_(C) for other groups.

### Crystal data

**Table:**

C_16_H_17_ClO	*F*(000) = 552
*M**_r_* = 260.75	*D*_x_ = 1.228 Mg m^−^^3^
Monoclinic, *P*2_1_/*n*	Mo *K*α radiation, λ = 0.71073 Å
*a* = 13.7630 (4) Å	Cell parameters from 3552 reflections
*b* = 6.0841 (2) Å	θ = 1.7–28.5°
*c* = 17.5003 (6) Å	µ = 0.26 mm^−^^1^
β = 105.726 (2)°	*T* = 293 K
*V* = 1410.54 (8) Å^3^	Block, colourless
*Z* = 4	0.30 × 0.25 × 0.20 mm

### Data collection

**Table:**

Bruker SMART APEXII area-detector diffractometer	3552 independent reflections
Radiation source: fine-focus sealed tube	2611 reflections with *I* > 2σ(*I*)
Graphite monochromator	*R*_int_ = 0.022
ω and φ scans	θ_max_ = 28.5°, θ_min_ = 1.7°
Absorption correction: multi-scan (*SADABS*; Bruker, 2008)	*h* = −17→18
*T*_min_ = 0.927, *T*_max_ = 0.951	*k* = −8→8
13158 measured reflections	*l* = −21→23

### Refinement

**Table:**

Refinement on *F*^2^	Primary atom site location: structure-invariant direct methods
Least-squares matrix: full	Secondary atom site location: difference Fourier map
*R*[*F*^2^ > 2σ(*F*^2^)] = 0.044	Hydrogen site location: inferred from neighbouring sites
*wR*(*F*^2^) = 0.130	H-atom parameters constrained
*S* = 1.03	*w* = 1/[σ^2^(*F*_o_^2^) + (0.0613*P*)^2^ + 0.2499*P*] where *P* = (*F*_o_^2^ + 2*F*_c_^2^)/3
3552 reflections	(Δ/σ)_max_ < 0.001
165 parameters	Δρ_max_ = 0.21 e Å^−^^3^
0 restraints	Δρ_min_ = −0.33 e Å^−^^3^

### Special details

**Table:**

Geometry. All e.s.d.'s (except the e.s.d. in the dihedral angle between two l.s. planes) are estimated using the full covariance matrix. The cell e.s.d.'s are taken into account individually in the estimation of e.s.d.'s in distances, angles and torsion angles; correlations between e.s.d.'s in cell parameters are only used when they are defined by crystal symmetry. An approximate (isotropic) treatment of cell e.s.d.'s is used for estimating e.s.d.'s involving l.s. planes.
Refinement. Refinement of *F*^2^ against ALL reflections. The weighted *R*-factor *wR* and goodness of fit *S* are based on *F*^2^, conventional *R*-factors *R* are based on *F*, with *F* set to zero for negative *F*^2^. The threshold expression of *F*^2^ > σ(*F*^2^) is used only for calculating *R*-factors(gt) *etc*. and is not relevant to the choice of reflections for refinement. *R*-factors based on *F*^2^ are statistically about twice as large as those based on *F*, and *R*- factors based on ALL data will be even larger.

### Fractional atomic coordinates and isotropic or equivalent isotropic displacement parameters (Å^2^)

**Table:**

	*x*	*y*	*z*	*U*_iso_*/*U*_eq_	
C1	0.25679 (10)	−0.3118 (3)	0.46901 (9)	0.0552 (4)	
C2	0.30278 (13)	−0.1202 (3)	0.50163 (9)	0.0631 (4)	
H2	0.3008	−0.0766	0.5521	0.076*	
C3	0.35172 (13)	0.0062 (3)	0.45851 (9)	0.0600 (4)	
H3	0.3831	0.1356	0.4807	0.072*	
C4	0.35569 (10)	−0.0537 (2)	0.38278 (8)	0.0490 (3)	
C5	0.30813 (11)	−0.2484 (3)	0.35197 (9)	0.0572 (4)	
H5	0.3095	−0.2927	0.3014	0.069*	
C6	0.25905 (12)	−0.3775 (3)	0.39444 (9)	0.0592 (4)	
H6	0.2278	−0.5076	0.3729	0.071*	
C7	0.40805 (11)	0.0754 (2)	0.33556 (8)	0.0518 (3)	
H7	0.4065	0.0202	0.2857	0.062*	
C8	0.45744 (11)	0.2628 (2)	0.35633 (8)	0.0531 (3)	
H8	0.4574	0.3211	0.4055	0.064*	
C9	0.51163 (10)	0.3859 (2)	0.30951 (8)	0.0471 (3)	
C10	0.56090 (11)	0.5706 (2)	0.33930 (8)	0.0529 (3)	
H10	0.5568	0.6189	0.3887	0.063*	
C11	0.62046 (10)	0.6992 (2)	0.29819 (8)	0.0505 (3)	
C12	0.63343 (11)	0.6046 (3)	0.22279 (9)	0.0575 (4)	
H12A	0.6915	0.5076	0.2353	0.069*	
H12B	0.6472	0.7230	0.1901	0.069*	
C13	0.54145 (11)	0.4765 (2)	0.17534 (8)	0.0508 (3)	
C14	0.51482 (11)	0.3025 (2)	0.22953 (8)	0.0527 (3)	
H14A	0.4494	0.2406	0.2030	0.063*	
H14B	0.5640	0.1847	0.2371	0.063*	
C15	0.56696 (15)	0.3622 (3)	0.10559 (10)	0.0780 (5)	
H15A	0.5103	0.2760	0.0771	0.117*	
H15B	0.6244	0.2682	0.1251	0.117*	
H15C	0.5823	0.4707	0.0707	0.117*	
C16	0.45296 (13)	0.6332 (3)	0.14473 (10)	0.0704 (5)	
H16A	0.4715	0.7444	0.1124	0.106*	
H16B	0.4358	0.7012	0.1889	0.106*	
H16C	0.3958	0.5527	0.1137	0.106*	
O1	0.65928 (9)	0.87306 (19)	0.32485 (7)	0.0688 (3)	
Cl1	0.19413 (4)	−0.47224 (9)	0.52256 (3)	0.08168 (19)	

### Atomic displacement parameters (Å^2^)

**Table:**

	*U*^11^	*U*^22^	*U*^33^	*U*^12^	*U*^13^	*U*^23^
C1	0.0494 (7)	0.0594 (9)	0.0550 (8)	−0.0006 (7)	0.0112 (6)	0.0115 (7)
C2	0.0715 (10)	0.0726 (11)	0.0450 (8)	−0.0080 (8)	0.0157 (7)	−0.0013 (7)
C3	0.0696 (9)	0.0585 (9)	0.0504 (8)	−0.0135 (7)	0.0139 (7)	−0.0063 (7)
C4	0.0477 (7)	0.0489 (8)	0.0500 (7)	0.0014 (6)	0.0124 (6)	0.0008 (6)
C5	0.0621 (8)	0.0566 (9)	0.0551 (8)	−0.0045 (7)	0.0192 (7)	−0.0078 (7)
C6	0.0598 (8)	0.0542 (9)	0.0627 (9)	−0.0087 (7)	0.0150 (7)	−0.0033 (7)
C7	0.0558 (8)	0.0525 (8)	0.0481 (7)	0.0004 (6)	0.0155 (6)	−0.0010 (6)
C8	0.0572 (8)	0.0558 (8)	0.0468 (7)	−0.0018 (7)	0.0150 (6)	−0.0011 (6)
C9	0.0470 (7)	0.0471 (7)	0.0458 (7)	0.0024 (6)	0.0100 (5)	0.0007 (6)
C10	0.0564 (8)	0.0568 (8)	0.0448 (7)	−0.0035 (7)	0.0126 (6)	−0.0069 (6)
C11	0.0441 (6)	0.0506 (8)	0.0526 (8)	−0.0005 (6)	0.0059 (6)	−0.0008 (6)
C12	0.0507 (8)	0.0647 (9)	0.0602 (9)	−0.0044 (7)	0.0206 (6)	−0.0052 (7)
C13	0.0545 (7)	0.0525 (8)	0.0464 (7)	0.0003 (6)	0.0155 (6)	−0.0027 (6)
C14	0.0625 (8)	0.0456 (7)	0.0499 (8)	−0.0012 (6)	0.0153 (6)	−0.0048 (6)
C15	0.0945 (13)	0.0874 (13)	0.0605 (10)	−0.0114 (10)	0.0356 (9)	−0.0178 (9)
C16	0.0686 (10)	0.0708 (11)	0.0614 (9)	0.0072 (9)	0.0000 (8)	0.0082 (8)
O1	0.0740 (7)	0.0596 (7)	0.0712 (7)	−0.0183 (6)	0.0170 (6)	−0.0110 (5)
Cl1	0.0819 (3)	0.0909 (4)	0.0745 (3)	−0.0193 (2)	0.0250 (2)	0.0201 (2)

### Geometric parameters (Å, º)

**Table:**

C1—C6	1.373 (2)	C10—C11	1.457 (2)
C1—C2	1.375 (2)	C10—H10	0.9300
C1—Cl1	1.7356 (15)	C11—O1	1.2192 (17)
C2—C3	1.375 (2)	C11—C12	1.494 (2)
C2—H2	0.9300	C12—C13	1.526 (2)
C3—C4	1.390 (2)	C12—H12A	0.9700
C3—H3	0.9300	C12—H12B	0.9700
C4—C5	1.390 (2)	C13—C16	1.525 (2)
C4—C7	1.463 (2)	C13—C15	1.526 (2)
C5—C6	1.378 (2)	C13—C14	1.531 (2)
C5—H5	0.9300	C14—H14A	0.9700
C6—H6	0.9300	C14—H14B	0.9700
C7—C8	1.327 (2)	C15—H15A	0.9600
C7—H7	0.9300	C15—H15B	0.9600
C8—C9	1.4560 (19)	C15—H15C	0.9600
C8—H8	0.9300	C16—H16A	0.9600
C9—C10	1.342 (2)	C16—H16B	0.9600
C9—C14	1.5006 (19)	C16—H16C	0.9600
			
C6—C1—C2	121.00 (14)	C10—C11—C12	116.55 (12)
C6—C1—Cl1	119.47 (12)	C11—C12—C13	113.41 (12)
C2—C1—Cl1	119.53 (12)	C11—C12—H12A	108.9
C3—C2—C1	119.04 (14)	C13—C12—H12A	108.9
C3—C2—H2	120.5	C11—C12—H12B	108.9
C1—C2—H2	120.5	C13—C12—H12B	108.9
C2—C3—C4	121.89 (14)	H12A—C12—H12B	107.7
C2—C3—H3	119.1	C16—C13—C15	109.80 (14)
C4—C3—H3	119.1	C16—C13—C12	109.72 (13)
C3—C4—C5	117.22 (13)	C15—C13—C12	109.33 (13)
C3—C4—C7	123.38 (13)	C16—C13—C14	110.34 (13)
C5—C4—C7	119.39 (13)	C15—C13—C14	109.04 (13)
C6—C5—C4	121.67 (14)	C12—C13—C14	108.58 (12)
C6—C5—H5	119.2	C9—C14—C13	114.60 (12)
C4—C5—H5	119.2	C9—C14—H14A	108.6
C1—C6—C5	119.18 (14)	C13—C14—H14A	108.6
C1—C6—H6	120.4	C9—C14—H14B	108.6
C5—C6—H6	120.4	C13—C14—H14B	108.6
C8—C7—C4	126.92 (14)	H14A—C14—H14B	107.6
C8—C7—H7	116.5	C13—C15—H15A	109.5
C4—C7—H7	116.5	C13—C15—H15B	109.5
C7—C8—C9	126.19 (14)	H15A—C15—H15B	109.5
C7—C8—H8	116.9	C13—C15—H15C	109.5
C9—C8—H8	116.9	H15A—C15—H15C	109.5
C10—C9—C8	119.59 (13)	H15B—C15—H15C	109.5
C10—C9—C14	120.39 (13)	C13—C16—H16A	109.5
C8—C9—C14	119.99 (12)	C13—C16—H16B	109.5
C9—C10—C11	123.40 (13)	H16A—C16—H16B	109.5
C9—C10—H10	118.3	C13—C16—H16C	109.5
C11—C10—H10	118.3	H16A—C16—H16C	109.5
O1—C11—C10	121.60 (14)	H16B—C16—H16C	109.5
O1—C11—C12	121.83 (14)		
			
C6—C1—C2—C3	−0.1 (2)	C8—C9—C10—C11	177.38 (13)
Cl1—C1—C2—C3	−179.65 (12)	C14—C9—C10—C11	−0.7 (2)
C1—C2—C3—C4	0.3 (3)	C9—C10—C11—O1	175.58 (14)
C2—C3—C4—C5	−0.2 (2)	C9—C10—C11—C12	−5.8 (2)
C2—C3—C4—C7	−179.28 (14)	O1—C11—C12—C13	−147.07 (14)
C3—C4—C5—C6	−0.1 (2)	C10—C11—C12—C13	34.32 (19)
C7—C4—C5—C6	179.07 (13)	C11—C12—C13—C16	66.81 (17)
C2—C1—C6—C5	−0.1 (2)	C11—C12—C13—C15	−172.71 (14)
Cl1—C1—C6—C5	179.41 (11)	C11—C12—C13—C14	−53.85 (17)
C4—C5—C6—C1	0.2 (2)	C10—C9—C14—C13	−21.6 (2)
C3—C4—C7—C8	−0.4 (2)	C8—C9—C14—C13	160.34 (12)
C5—C4—C7—C8	−179.51 (14)	C16—C13—C14—C9	−72.87 (16)
C4—C7—C8—C9	178.10 (13)	C15—C13—C14—C9	166.46 (13)
C7—C8—C9—C10	−178.00 (15)	C12—C13—C14—C9	47.41 (16)
C7—C8—C9—C14	0.1 (2)		

### Hydrogen-bond geometry (Å, º)

**Table:**

*D*—H···*A*	*D*—H	H···*A*	*D*···*A*	*D*—H···*A*
C2—H2···O1^i^	0.93	2.41	3.301 (2)	159
C14—H14*B*···O1^ii^	0.97	2.57	3.4299 (18)	148

Symmetry codes: (i) −*x*+1, −*y*+1, −*z*+1; (ii) *x*, *y*−1, *z*.
